# Modelling the interplay between responsive individual vaccination decisions and the spread of SARS-CoV-2

**DOI:** 10.1016/j.epidem.2025.100831

**Published:** 2025-04-23

**Authors:** Karina Wallrafen-Sam, Maria Garcia Quesada, Benjamin A. Lopman, Samuel M. Jenness

**Affiliations:** a Life & Medical Sciences Institute (LIMES) and Bonn Center for Mathematical Life Sciences, University of Bonn, Bonn, Germany; b Department of Epidemiology, Rollins School of Public Health, Emory University, Atlanta, GA, USA

**Keywords:** SARS-CoV-2, Agent-based model, Network-based model, Vaccination decision-making, Vaccine hesitancy

## Abstract

**Background:**

COVID-19 vaccine hesitancy proved to be a major barrier to higher uptake, but it is unclear whether interventions targeting hesitancy could result in substantial prevention benefits. Epidemic models that represent vaccine decision-making psychology can provide insight into the potential impact of vaccine promotion interventions in the context of the COVID-19 pandemic and future epidemics of vaccine-preventable diseases.

**Methods:**

We coupled a network- and agent-based model of SARS-CoV-2 transmission with a social-psychological vaccination decision-making model in which vaccine side effects and breakthrough infections could “nudge” individuals towards vaccine resistance while spikes in COVID-19 hospitalizations could nudge them towards vaccine willingness. This model was parameterized and calibrated to represent the COVID-19 epidemic in Georgia, USA from January 2021 to August 2022. We modelled counterfactual scenarios in which increases to resistant-to-willing nudges were combined with decreases to willing-to-resistant nudges. We compared cumulative vaccine doses administered, SARS-CoV-2 incidence, and COVID-related deaths across scenarios.

**Results:**

Increasing the probability of hospitalization-prompted resistant-to-willing nudges increased vaccine uptake by as much as 5.4 % and decreased SARS-CoV-2 incidence by as much as 4.0 %. In contrast, decreasing the probability of breakthrough infection-related willing-to-resistant nudges had a negligible impact on further vaccination and disease outcomes.

**Conclusions:**

Vaccine promotion interventions that address community-level factors influencing decision-making may have a greater ability to avert SARS-CoV-2 infections than those targeted to individual vaccination and infection history. Additionally, reactive vaccine promotion interventions may have only limited prevention benefits in the short term, suggesting that attention should be paid to formulating interventions that accurately anticipate the case curve.

## 1. Background

The most impactful intervention to date against the COVID-19 global pandemic has been the development and distribution of highly effective vaccines (Suthar et al., 2022; Baden et al., 2021). The COVID-19 vaccine rollout in the United States began in late 2020 with a two-dose primary series, before waning immunity and decreased effectiveness against novel SARS-CoV-2 strains prompted the development of four sequential booster vaccinations (Moulia et al., 2023). Although these vaccines have been widely available in the United States since 2021, only about 70 % of the eligible U.S. population had completed the primary vaccine series as of mid-2023 (Centers for Disease Control and Prevention, 2021a). Primary series coverage was even lower in the state of Georgia, at around 58 % overall with lower uptake in rural counties and among racial minority groups (Centers for Disease Control and Prevention, 2021a; Saelee et al., 2022; Medcalfe and Slade, 2023). Vaccine uptake slowed in the later months of 2021, has been consistently lower in younger age groups, and tapered off markedly for subsequent doses (Centers for Disease Control and Prevention, 2021a). This is partially due to vaccine hesitancy – a complex phenomenon related to concerns about the safety, efficacy, and necessity of these vaccines (Bendetson et al., 2023; Dubé et al., 2021).

Encouraging higher vaccine uptake by addressing vaccine hesitancy is crucial, but the effects of vaccine promotion interventions can be difficult to predict since decisions on whether and when to receive a vaccine dose are influenced by a multitude of factors – including fear of illness (Aguolu et al., 2022), altruism (Aguolu et al., 2022; Cucciniello et al., 2022), social conformity (Hamel et al., 2021; Leong et al., 2022), and information spread via social contacts, news outlets, or social media (Yadete et al., 2021; de Albuquerque Veloso Machado et al., 2021). Agent-based mathematical models can compare counterfactual scenarios and represent complex individual- and community-level behaviours, providing insight into the optimal formulation and timing of such interventions. While several models of vaccination decision-making exist, most of them consider one-time decisions; are focused on theory rather than real-world scenarios; and, most crucially, are rooted in game theory, meaning they rely on the assumption of rational actors (Bai, 2021; Becchetti et al., 2021; Deka et al., 2020; Xia and Liu, 2014). In contrast, social psychological research suggests that individuals typically rely on heuristics rather than rational cost-benefit analyses when making complex decisions (Tversky and Kahneman, 1974). For example, decisions generally obey the law of inertia: they tend to remain stable over time but are sensitive to small “nudges” from unfavourable outcomes (Thaler and Sunstein, 2008). To provide valid insights into vaccination interventions, agent-based models are needed that account for such heuristics and capture feedback effects between behaviour and epidemic outcomes.

The role of heuristics and inertia in decision-making has been studied in the context of annual influenza vaccination but is not yet well understood in the context of the recent COVID-19 vaccine rollout. Papst et al. developed a seasonal influenza model in which prior infections and vaccine side effects could “nudge” persons to change their future vaccination decisions, creating a feedback loop between behaviour and disease spread (Papst et al., 2022). This model, while theoretical in its focus, was consistent with the empirical findings of a longitudinal cohort study on trends in influenza vaccination: the cohort’s vaccination behaviour was generally stable over time, but flu infections could influence subsequent vaccination decisions and those who did switch between approaches tended to persist in their new behaviour, in a practical illustration of the law of inertia (Walsh et al., 2020). While equivalent longitudinal analyses are not yet available for COVID-19, recent studies have demonstrated the role of an inertia-like “status quo bias” – i.e., the tendency to maintain current behaviors – in COVID-19 vaccination decision-making (Tentori et al., 2022) and established that past vaccination behavior is predictive of future vaccination intentions (Luisi et al., 2024). Similar studies have also distinguished stable vaccine acceptors and non-acceptors from movable individuals who can be “nudged” by factors such as vaccine side effects or social pressure (Zimmermann et al., 2023; Hyland et al., 2021). As such, adapting the modelling framework developed by Papst et al. for an agent-based mathematical model of SARS-CoV-2 could provide insight into how vaccine promotion interventions could have been formulated and targeted specifically to boost the stagnant levels of COVID-19 vaccine coverage.

In this study, we utilized a high-resolution agent-based model of SARS-CoV-2 dynamics in the state of Georgia – a high-burden population with particularly low vaccine coverage – and coupled it with a social-psychological decision-making model in which vaccine side effects, breakthrough infections, and the overall state of the outbreak could “nudge” individual agents to change their vaccination behaviour. Our goal was to use our novel combination of methods to explore how changes to the probabilities of individuals adapting their behaviour after experiencing one of these “nudges” might impact disease incidence.

## 2. Methods

This study used a network- and agent-based model of SARS-CoV-2 transmission, disease progression, and vaccination behaviours in the population of Georgia, USA over a twenty-month period from January 2021 to August 2022 – i.e., the month in which eligibility for the first vaccine dose began to expand in Georgia to the month before the start of the bivalent booster rollout. Our model was built using EpiModelCOVID, a previously validated extension of the EpiModel software platform, which uses the statistical framework of exponential random graph models (ERGMs) to simulate dynamic contact networks (Jenness et al., 2018). For this study, we built a social-psychological decision-making model into the vaccination processes within EpiModelCOVID. The model code and software are available on GitHub (https://github.com/EpiModel/COVID-Vax-Decisions).

### Core Model Structure.

Our model tracked 100,000 persons (agents) representing a sampled population of the entire state of Georgia, USA, without accounting for regional variations. Agents were assigned an initial age according to Georgia’s age pyramid as of 2020 (National Center for Health Statistics, 2021). They could exit the model population at any time through death (general or disease-specific), while new agents entered the model population exclusively through birth. All modelled agents were members of two distinct, overlapping contact network layers and transmission environments: community and household.

For the community network layer, all contacts (edges) were assumed to be non-persistent (no duration). Based on the COVIDVu study, we estimated the mean daily degree for this network layer to be 13.8 across all agents and 5.7 across agents aged 65 years or older (Nelson et al., 2022). The POLYMOD social mixing study extrapolated to U.S. settings was used to parameterize age mixing, with the within-group contact proportion set at 69 % for those aged under 18 years, 81 % for 18- to 64-year-olds, and 21 % for 65 + -year-olds (Prem et al., 2017). The community environment was estimated with an ERGM from which we then simulated at each timestep.

The household network layer was comprised of persistent contacts, lasting from entry into the population to simulation end or death. Each agent was assigned to a household according to an algorithm based on U. S. Census data: 1) 29.2 % of households had at least one member aged under 18 years (U.S. Census Bureau, 2020); 2) 79.1 % of households had at least one 18- to 64-year-old (U.S. Census Bureau, 2020); 3) 31.4 % of households had at least one 65 + -year-old (U.S. Census Bureau, 2020); 4) the average household had 2.7 persons (U.S. Census Bureau, 2021a); 5) every household with a child also had at least one adult; and 6) 97.9 % of children had an 18- to 64-year-old in their household (U.S. Census Bureau, 2021b). We took this approach due to the lack of recent social mixing data for children in U.S. settings. Household edges were specified such that each household was fully saturated and each edge was within a single household. Community and household contacts were subsequently combined to create a multi-layer dynamic network.

Our model represented the natural history of COVID-19 using a SEIRS framework. Susceptible agents could stochastically transition to the exposed state upon contact with an infected person (i.e., a discordant contact). The daily probability of infection given a discordant contact depended on the vaccination status of the susceptible agent, the symptom status of the infectious agent, and whether the contact was household- or community-level. Newly infected agents were stochastically assigned to either an asymptomatic or symptomatic clinical pathway, with a subset of symptomatic agents subsequently designated for hospitalization. The probabilities of symptoms and hospitalization both depended on age and vaccination status. Agents in the hospitalized state experienced a higher age-specific mortality rate than those in other states. Once recovered, agents stochastically re-entered the susceptible state, where they could be reinfected. Parameters defining the model’s disease progression, transmission, and clinical epidemiology (Table 1) were either drawn from existing literature or calibrated as described below.

### Vaccination Decision-Making Process.

Each adult agent was assigned a binary vaccination “type”– resistant or willing – such that the prevalence of vaccine willingness by age group at the start of the vaccine rollout matched the empirical distribution in late 2020 (Kelly et al., 2021; Nikolovski et al., 2021). The vaccination decision-making process for agents aged under 18 years was not explicitly modelled, given fundamental differences in this process for minors versus adults. Instead, children received vaccine doses according to age-specific rates.

In accordance with the law of inertia in decision-making (Thaler and Sunstein, 2008), agents maintained their initial attitude toward vaccination until an adverse event (“nudge”) prompted them to change – meaning disease outcomes could affect vaccination behaviour, which in turn affected future disease outcomes. Three such nudges were considered: 1) experiencing vaccine side effects could prompt a vaccine willing individual (who had received at least one dose) to become vaccine resistant; 2) experiencing an infection while fully vaccinated (a “breakthrough infection”) could prompt a vaccine willing individual (who had received at least two doses) to lose trust in the vaccine and become vaccine resistant; and 3) increased hospitalized prevalence in the population could prompt any vaccine resistant individual to grow more concerned about the spread of COVID-19 and become vaccine willing. For model calibration purposes, an additional willing-to-resistant pathway was included to cover all other reasons for developing vaccine resistant attitudes among individuals who had received at least one dose, including (but not limited to) social conformity (Leong et al., 2022), social contacts’ negative experiences with vaccines (Quon et al., 2023), the spread of information via news outlets and social media (de Albuquerque Veloso Machado et al., 2021; Ruiz and Bell, 2021), and the general phenomenon of “pandemic fatigue” – i. e., a decreased willingness to engage in protective behaviours against COVID-19 among individuals who no longer view it as a novel threat (Lazarus et al., 2024).

To parameterize the first two nudges, we identified: 1) the odds ratio comparing the likelihood of booster willingness for those who had versus had not missed work due to side effects from the primary vaccination series (Chrissian et al., 2022); and 2) the odds ratio comparing the likelihood of booster willingness for those who had received the primary vaccination series and had versus had not been subsequently infected (Dziedzic et al., 2022). We converted these odds ratios to one-time probabilities of being “nudged” towards vaccine resistance. For the third nudge, we estimated the probability that a vaccine-eligible adult in Georgia was convinced to vaccinate by an increase in hospitalized COVID-19 prevalence between July and September 2021, using the finding that 38 % of that period’s surveyed late adopters were motivated by concern about local hospitalizations (Hamel et al., 2021).

At any timestep, agents could stochastically undergo vaccination if they were not currently symptomatic, had not tested positive in the last two weeks, were vaccine willing (for adult agents), and were currently eligible for their next dose based on their age group and vaccination history. Vaccination reduced the risk of disease acquisition, the risk of progression to symptomatic disease, and the risk of eventual hospitalization via a “leaky” mechanism. Vaccine immunity waned over time following an exponential decay with a half-life of 80 days (Centers for Disease Control and Prevention, 2021b).

### Calibration.

We used an adapted Approximate Bayesian Computation (ABC) approach for model calibration. We defined uniform prior distributions for uncertain model parameters and identified corresponding target statistics; sampled initial parameters via a grid search approach; and selected the best fit parameters using numerical distance minimization between simulation results and corresponding targets, combined with visual inspection of the fitted model against empirical data. To account for model stochasticity, we performed ten simulations per parameter set considered.

Specifically, the per-contact infection probability was calibrated so that the simulated number of incident infections by month matched the confirmed case counts reported by the Georgia Department of Public Health (GDPH) (Georgia Department of Public Health), adjusted to account for underreporting (Institute for Health Metrics and Evaluation, University of Washington, 2022) and scaled to a population of 100,000. To account for time-varying coverage of non-pharmaceutical interventions and the introduction of new variants, this per-contact infection probability was boosted by 34 % for two periods of increased transmission (from 01 November 2020–15 January 2021 and from 01 December 2021–31 January 2022) and suppressed by 40 % for three periods of decreased transmission (from 16 January 2021–29 June 2021, from 15 September 2021–30 November 2021, and from 01 February 2022 onward). The age-specific hospitalization proportions and disease-related mortality multiplier were calibrated so that the resultant COVID-19 related hospital admissions and deaths by month matched GDPH reports (Georgia Department of Public Health). Finally, age- and dose-specific vaccination rates and the nudge probabilities for the additional willing-to-resistant pathway were calibrated so that the resultant vaccine coverage by age, dose, and month matched the levels reported by the CDC for Georgia (Centers for Disease Control and Prevention, 2021a). The full list of prior distributions and target statistics is available in Section 7 of the Supplemental Appendix.

### Intervention Scenarios.

We compared our calibrated model to counterfactual scenarios that explored hypothetical interventions on two of the three nudges in our model. These interventions did not affect the precipitating events, but rather the agents’ responses to them. Specifically, we 1) kept the probability that vaccine side effects would prompt a vaccine willing-to-resistant switch constant across all scenarios; 2) decreased the probability that a breakthrough infection would prompt a willing-to-resistant switch (the Breakthrough Nudge Probability or Breakthrough NP) to as little as zero; and 3) increased the probability that a spike in hospitalizations would prompt a resistant-to-willing switch (the Hospitalization Nudge Probability or Hospitalization NP) to as much as two times the reference value. We then also explored the impact of applying these changes to only older adults (65 +-year-olds) or only younger adults (18- to 64-year-olds) instead of all adult agents.

Since data on the duration of COVID-19 immunity was limited, we performed sensitivity analyses on two particularly uncertain parameters: the average duration of natural immunity to infection after recovery and the half-life of vaccine-induced immunity.

### Model Output.

For each model scenario, we tracked the incidence rate of SARS-CoV-2 infections, the COVID-related death rate, and the per-dose vaccine coverage by age group over time. The median and 50 % simulation interval (SI) of each output across 128 simulations per scenario were reported.

## 3. Results

### Model Overview.

**Fig. 1.**
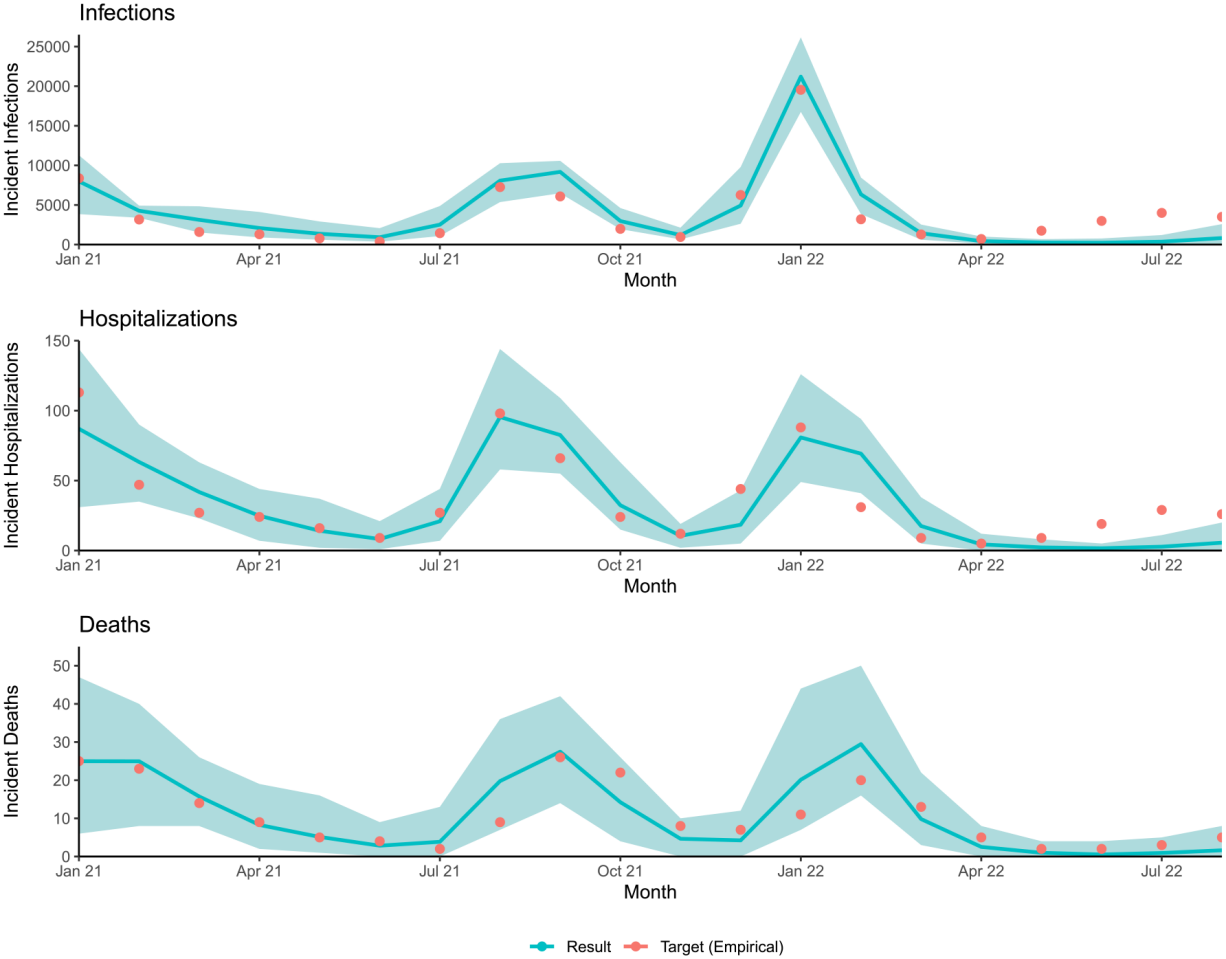
Model calibration results for cases, hospitalizations, and deaths. The reference model was calibrated to (1) an estimate of the total number of incident SARS-CoV-2 infections, (2) the reported number of confirmed COVID-19-related hospital admissions, and (3) the reported number of confirmed COVID-19-related deaths, all per 100,000 persons in Georgia per month.

**Fig. 2.**
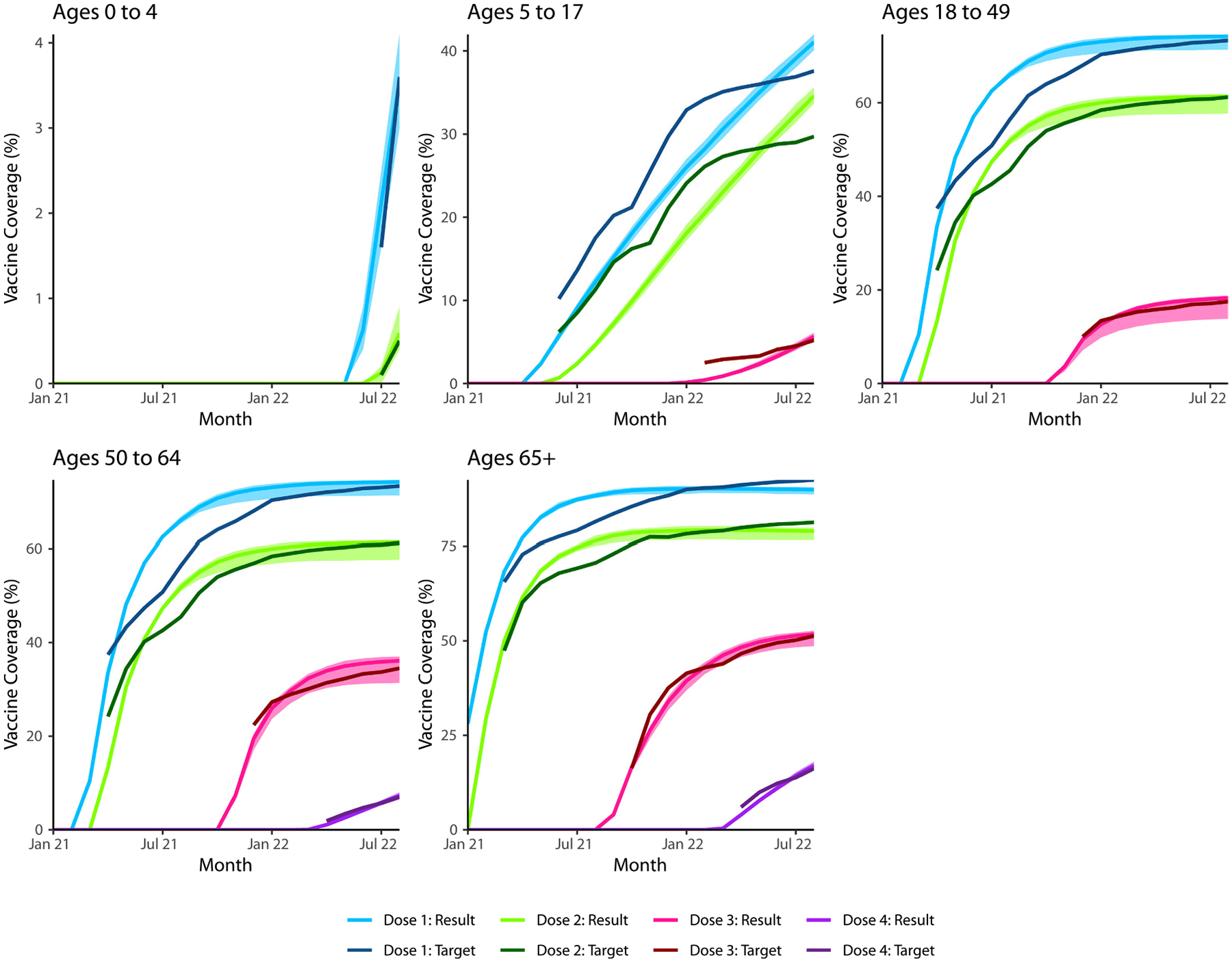
Model calibration results for vaccine coverage. The reference model was also calibrated to the reported vaccine coverage levels in Georgia by age group, dose, and month.

Fig. 1 shows the calibrated model’s simulated monthly infection, hospitalization, and death rates against empirical trends in Georgia, with hospitalizations peaking in August 2021 (Delta wave) and infections peaking in January 2022 (Omicron wave). There were 131.1 infections (50 % SI: 129.9, 132.3), 82.0 symptomatic cases (50 % SI: 81.2, 82.7), and 0.36 deaths (50 % SI: 0.35, 0.38) per 100,000 person-days in this model. Fig. 2 displays the model’s simulated vaccine coverage by age group and dose against the corresponding empirical coverage.

### Intervention Impacts.

**Fig. 3.**
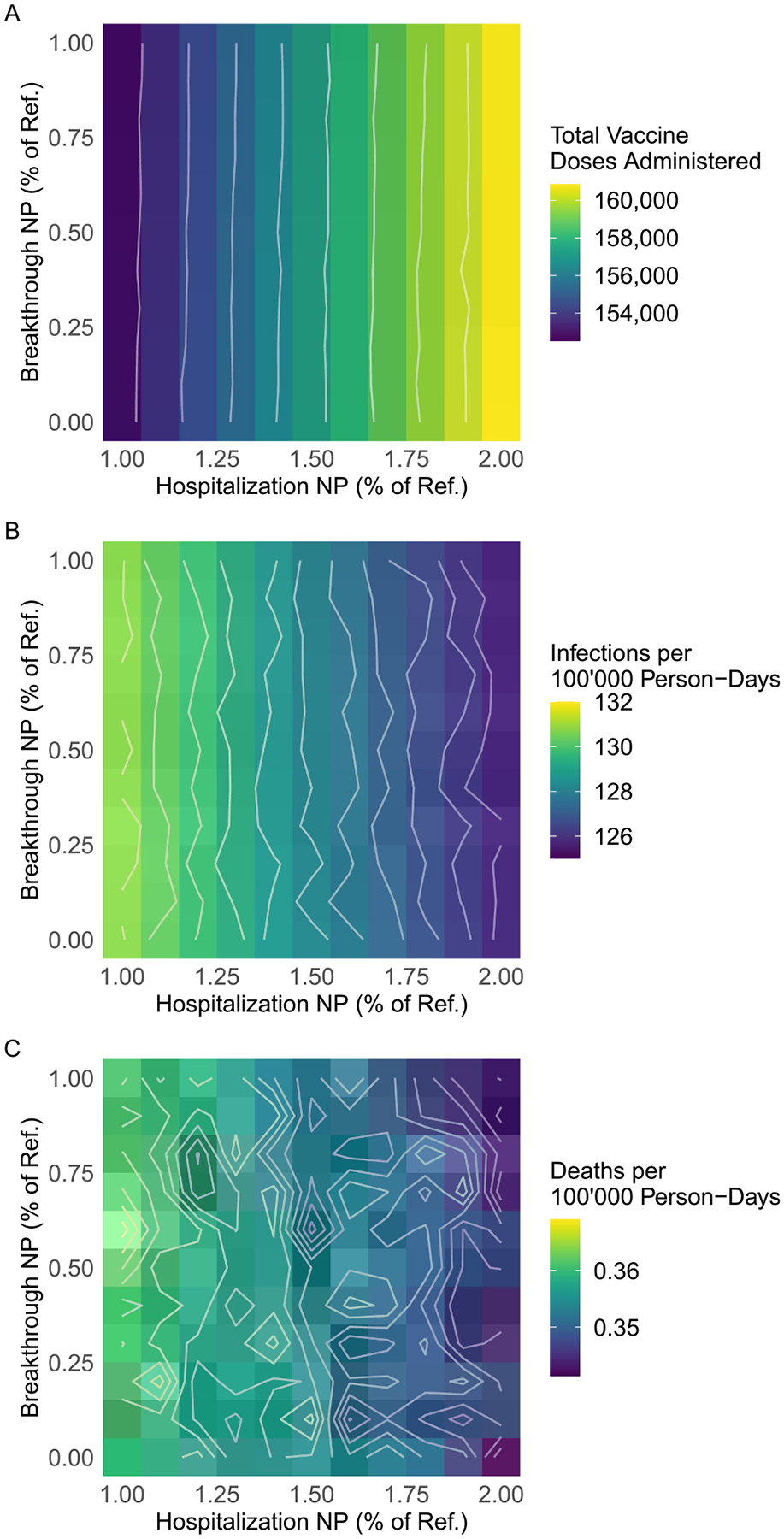
Vaccines administered, incidence rate, and death rate by model scenario. The hospitalization nudge probability (Hospitalization NP) was increased from 100 % to 200 % of its reference value and the breakthrough nudge probability (Breakthrough NP) was decreased from 100 % to 0 % of its reference value, both in increments of 10 %. For each parameter combination, the median number of vaccine doses administered per run, the median infection rate per 100,000 person-days, and the median disease-related death rate per 100,000 person-days across 128 runs are displayed.

Doubling the Hospitalization NP generally led to a slight increase in the coverage of all vaccine doses; the absolute change ranged from about 1–5 % (for example, for 18- to 49-year-olds, coverage changed from 74.2 %, 61.4 %, and 18.3 % for the first, second, and third doses to 77.0 %, 64.9 %, and 22.8 %). Reducing the Breakthrough NP to 0 had no discernible impact (Table 2, Fig. 3A).

The overall pattern for incidence was the inverse of that for doses: a higher Hospitalization NP generally led to fewer infections (Table 3 and Fig. 3B). The minimum incidence rate across scenarios was 125.6 infections per 100,000 person-days (50 % SI: 124.5, 127.3), corresponding to 5.5 (50 % SI: 3.8, 6.5) infections averted per 100,000 person-days compared to the reference scenario; this occurred when the Hospitalization NP was doubled and the Breakthrough NP was 40 % of its reference value. The minimum symptomatic case rate across scenarios was 77.8 cases per 100,000 person-days (50 % SI: 77.1, 78.7), corresponding to 4.2 (50 % SI: 3.3, 4.9) cases averted per 100,000 person-days compared to the reference scenario; this occurred when the Hospitalization NP was doubled and the Breakthrough NP was at its reference value.

Table 4 and Fig. 3C demonstrate a similar pattern for deaths, with more noise from model stochasticity. The minimum death rate across scenarios was 0.34 deaths per 100,000 person-days (50 % SI: 0.33, 0.36), corresponding to 0.02 deaths averted per 100,000 person-days (50 % SI: 0.00, 0.04) compared to the reference scenario; this occurred when the Hospitalization NP was doubled and the Breakthrough NP was 90 % of its reference value.

**Fig. 4.**
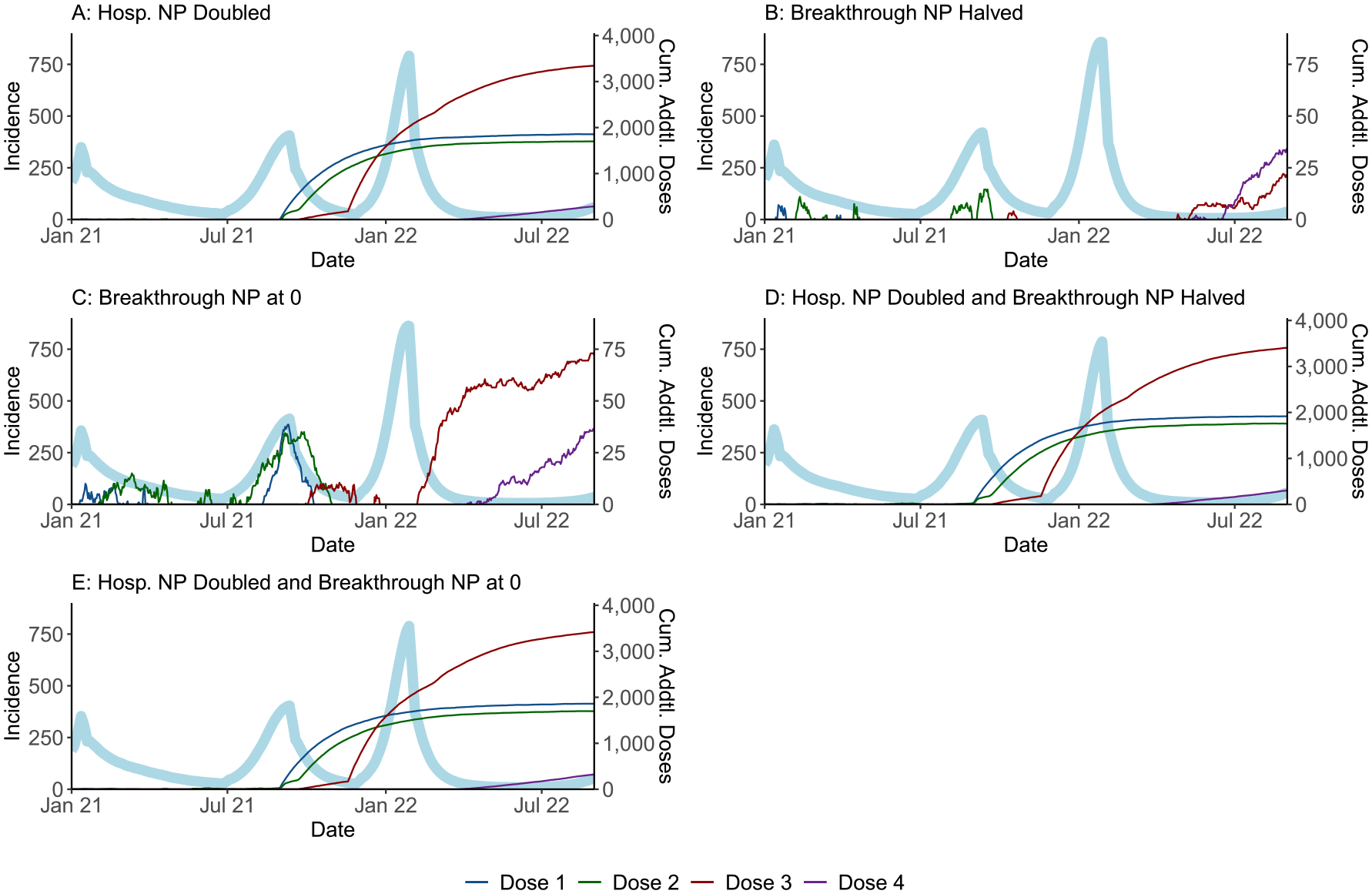
Epidemic curve vs. cumulative vaccines administered over time for select scenarios. The light blue lines represent the (median) incident infections by day across the full population of 100,000 nodes. The coloured lines represent the difference between the cumulative number (median) of vaccine doses administered across all age groups in the scenario of interest and the corresponding (median) number from the reference scenario, as of each point in time. Note that the secondary y-axes differ across panels.

Fig. 4 shows the impact of five selected intervention scenarios on vaccine coverage over time relative to the epidemic curve. Doubling the Hospitalization NP alone (Panel A) affected all vaccine-resistant persons and increased their vaccine uptake relative to the reference scenario across all doses, starting when the hospitalization threshold was typically crossed in late August 2021. As a result, the subsequent January 2022 infection peak was somewhat reduced. Halving or eliminating the Breakthrough NP (Panels B and C) affected only the much smaller population subset who experienced breakthrough infections and caused a nominal increase in their third- and fourth-dose uptake from February 2022 onward.

### Additional Results.

The relative impact of the different intervention scenarios was the same across age targeting approaches, but the interventions acted on a smaller scale when they were limited to those aged 65 + years (Supplemental Fig. S1 and S2). Sensitivity analyses of the infection- and vaccine-induced immunity parameters showed that, while the scale of the outbreak varied, the relative impact of three selected interventions was similar, except that interventions on the Hospitalization NP became less impactful when the half-life of vaccine induced immunity was increased from 80 days to 140 days or more (Supplemental Tables S1 and S2).

## 4. Discussion

In our study, we used the novel combination of a network-based model of SARS-CoV-2 dynamics and a social-psychological model of COVID-19 vaccination decision-making to investigate which of the pathways between vaccine willingness and resistance might have the greatest influence on vaccine coverage and disease incidence. Our detailed agent-based modelling framework allowed us to simulate individual contact patterns and decision-making while accounting for the interactions between individual attributes like age, household structure, vaccination history, and past experiences of infections. We found that doubling the probability that a spike in hospitalized prevalence would prompt vaccine hesitant persons to vaccinate increased vaccine uptake by 5.4 % and decreased incidence by 4.0 %, a small but still substantial impact. In contrast, eliminating the probability that a breakthrough infection would prompt a previously vaccine willing person to forgo further vaccination had negligible population-level benefits. Overall, our study suggests that epidemic outcomes are improved when as many vaccine-hesitant persons as possible are reached via interventions targeting community-level factors influencing their decision-making, and that optimizing the timing of any vaccine promotion intervention relative to the timing of infection waves – so that the interventions anticipate the case curve instead of reacting to it – is critical.

In our study, interventions affecting persons’ responses to a community-level factor – i.e., hospitalised prevalence – were impactful, while those affecting responses to an individual-level factor – i.e., a breakthrough infection – were not, primarily because far more persons were exposed to the former set of interventions than the latter. The hospitalization-related resistant-to-willing-pathway could affect anyone who was vaccine resistant when hospitalizations spiked – not just those who were hospitalized themselves. Conversely, the breakthrough-related willing-to-resistant pathway could only affect the small number of fully vaccinated and still vaccine willing individuals who became infected and experienced symptoms despite the layers of protection against both transmission and symptomatic disease provided by the vaccine. Thus, the fundamental difference between changes to the hospitalization nudge probability, which were beneficial on a population level, and changes to the breakthrough nudge probability, which were not, is that the former set of changes had a much wider reach. This finding regarding the importance of scale is consistent with empirical evaluations of vaccine promotion interventions: Athey et al. found that the impact of social media advertisements promoting COVID-19 vaccination was small on a per-person basis but in aggregate convinced upwards of 11 million persons (Athey et al., 2023). The distinction we made between individual- and community-level precipitating events is unique to our model. In the Papst et al. compartment model of influenza vaccination, segments of the population based their decisions solely on personal past experiences of infections or vaccine side effects; the two model parameters controlling these events (the vaccine efficacy and the probability of vaccine morbidity) were both relevant to their model predictions (Papst et al., 2022). By using an agent-based model that incorporates more varied precipitating events, our study contributes an additional finding: vaccine promotion interventions that focus on experiences shared by wide segments of the population may have more disease prevention potential than those focused on individual experiences with the COVID-19 vaccine in isolation.

Our results also suggest that an intervention’s disease prevention potential is significantly limited by suboptimal timing relative to the timing of infection waves. While all the interventions we tested were reactive to naturally occurring transmission events, changes to the hospitalization nudge probability were faster-acting than changes to the breakthrough nudge probability and therefore had a non-negligible impact on vaccine coverage and infections. Specifically, changes to the hospitalization nudge probability could affect the later of the two infection peaks within our model timeframe but not the earlier one, which was already well underway by the time the hospitalized prevalence in our model usually crossed its threshold in late August 2021. Meanwhile, the minor increases in third- and fourth-dose uptake caused by changes to the breakthrough nudge probability began very late in the model timeframe, by which point both infection peaks had passed and the number of infections that could potentially have been averted was small. These results are consistent with those of other COVID-19 modelling studies that have found the success of a particular vaccination campaign to be highly sensitive to its timing: Gavish et al. projected that advancing Israel’s summer 2021 booster campaign by 2 weeks could have halved the number of cases in the subsequent three months (Gavish et al., 2022). The models developed by Papst et al. and a preceding influenza study by Wells et al. both assumed that each annual influenza vaccine rollout concluded before that year’s outbreak began (Papst et al., 2022; Wells and Bauch, 2012); they therefore did not consider how the rollout’s impact could vary based on timing. Our model’s ability to account for concurrent vaccination and transmission dynamics facilitated our finding that proactively timed interventions – for example, using surveillance data and predictive modelling to identify an upcoming increase in hospitalisations and making it the focus of an information campaign encouraging vaccinations to prevent it, instead of a reactive campaign launched once the increase has begun – could have a greater impact on epidemic outcomes.

Finally, we found that limiting vaccine promotion interventions to those aged 65 years or older, who are at increased risk of severe outcomes, led to fewer prevention benefits than when either 18- to 64-year-olds or all adults were targeted. This was likely due in part to the relatively small size of the older age group and to their higher reference levels of vaccine willingness, which left less room for intervention-prompted increases. Our study supports findings from previous modelling studies suggesting that vaccine hesitancy should be addressed in younger populations, who are less susceptible to severe disease or death but who play a larger role in transmission and have lower baseline vaccine willingness (Gavish et al., 2022; Gog et al., 2021; Kim et al., 2022).

## 5. Limitations

One limitation of our approach is that we only explicitly modelled and tracked three factors that could affect vaccination decisions: vaccine side effects, breakthrough infections, and hospitalized prevalence. There may be many others, including both individual- and community-level factors that play an even larger role in vaccination decision-making than the ones we considered. For example, Lazarus et al. attributed a 21.8 percentage-point decrease in booster willingness over a one-year period to pandemic fatigue (Lazarus et al., 2024), while Ruiz et al. found a 19.1 percentage-point difference in vaccine acceptance between viewers of liberal versus conservative news channels (Ruiz and Bell, 2021). Future work on this model could explore the relative importance of these potential additional willing-to-resistant pathways.

We also did not consider clustering by vaccination type, correlations between a parent’s type and their children’s vaccination status, or the ways that vaccine resistant views might propagate through a network (e. g., how persons might be influenced by breakthrough infections within their household or social network even if they themselves were not a breakthrough case). By assuming that unvaccinated persons were distributed randomly through the network, we may have overestimated the indirect protection they received via vaccinated contacts. This may, in turn, have muted the impact of our interventions, since we did not account for the possibility of the interventions breaking up pockets of vaccine resistance within certain households or neighbourhoods – but as the issue of clustering is complex, it should be explored further by future work on this model.

Several key sociodemographic factors affecting vaccination decision-making have yet to be incorporated into our model — including race/ethnicity (Medcalfe and Slade, 2023), region (Saelee et al., 2022), and job sector (Purvis et al., 2022) — despite their relevance to the diverse population of Georgia. As such, we overlooked the role that employer vaccine mandates may play for healthcare workers, for example, and crucially, we cannot currently provide insights into the important issue of racial disparities in vaccine uptake.

Another limitation of our model is the uncertainty to which some of our parameters are subject. Due to data limitations, our reference willing-to-resistant nudge probabilities were extrapolated from surveys of vaccine willingness beyond their intended use. Since COVID-19 emerged relatively recently, data on the waning of vaccine-induced and natural immunity is currently limited, and we did not account for heterogeneity in immunity by age, disease severity, or other factors (Chen et al., 2021; Hussain et al., 2020; Wilk et al., 2020). To address this limitation, we performed sensitivity analyses on the two immunity duration parameters and found that our conclusions about which interventions were most effective generally held.

## 6. Conclusions

Our findings indicate that addressing community-level factors influencing decision-making may have more disease prevention potential than intervening based on individuals’ own vaccination and infection history, and that attention should be paid to timing the implementation of vaccination strategies such that they pre-empt increases in the case curve. These conclusions were facilitated by an agent-based model that included realistic details of human behaviour based on established results in social psychology, illustrating that models with greater psychological realism can be useful for informing future public health interventions that address barriers to vaccination.

## Supplementary Material

1

## Figures and Tables

**Table 1:** Selected model parameters.

Parameter	Value	Source
**Population & Household Characteristics**		
*Population proportion by age group* *	0.233, 0.620, 0.147	(National Center for Health Statistics, 2021)
*Avg. household size*	2.7	(U.S. Census Bureau, 2021a)
*Proportion of households with members in given age group* *	0.292, 0.791, 0.314	(U.S. Census Bureau, 2020)
*Proportion of children living with adult under 65*	0.979	(U.S. Census Bureau, 2021b)
**Community Contact Patterns**		
*Overall daily mean degree*	13.8	(Nelson et al., 2022)
*Daily mean degree for persons aged 65* +	5.7	(Nelson et al., 2022)
*Associative mixing proportion by age group* *	0.69, 0.81, 0.21	(Prem et al., 2017)
**Transmission & Natural History**		
*Reference per-contact transmission probability*	0.050	Calibrated
*Relative transmission risk if asymptomatic*	0.5	(Davies et al., 2020)
*Contacts per household pairing per day*	3	Calculated from (Madewell et al., 2022)
*Proportion symptomatic (incl. mildly symptomatic) by age group* ^	0.573, 0.642, 0.760, 0.800, 0.813, 0.814, 0.769, 0.723, 0.666	(Harrington, 2022)
*Proportion hospitalized by age group* ^	0.006, 0.006, 0.008, 0.015, 0.021, 0.027, 0.036, 0.046, 0.054	Calibrated; ratios from (Harrington, 2022)
*Avg. duration of latent period, in days*	5.5	(Xin et al., 2022)
*Avg. duration of pre-clinical infection, in days*	1.5	(Davies et al., 2020)
*Avg. duration of clinical infection, in days*	3.5	(Davies et al., 2020)
*Avg. duration of hospitalization, in days*	10.0	(Conlon et al., 2021)
*Avg. duration of asymptomatic infection, in days*	5.0	(Davies et al., 2020)
*Avg. duration of natural immunity, in days*	300	(Centers for Disease Control and Prevention, 2021b)
*General annual mortality rate, in deaths per 100,000 persons per year* ^†^	608, 30, 13, 22, 63, 116, 143, 187, 228, 300, 416, 600, 945, 1453, 1952, 2817, 4369, 7159, 15,626	(Georgia Department of Public Health)
*COVID-related mortality multiplier*	1800	Calibrated
**Vaccination**		
*Proportion initially vaccine willing* ^‡^	N/A, N/A, 0.70, 0.75, 0.91	(Kelly et al., 2021; Nikolovski et al., 2021)
*Hospitalization nudge probability*	0.102	Calculated from (Hamel et al., 2021)
*Side effect nudge probability*	0.073	(Chrissian et al., 2022)
*Breakthrough infection nudge probability*	0.125	(Dziedzic et al., 2022)
*Misc. nudge probability (post Dose 1)* ^‡^	N/A, N/A, 0.18, 0.18, 0.12	Calibrated
*Misc. nudge probability (post Dose 2)* ^‡^	N/A, N/A, 0.75, 0.43, 0.35	Calibrated
*Misc. nudge probability (post Dose 3)* ^‡^	N/A, N/A, N/A, 0.50, 0.42	Calibrated
*Hospitalization threshold in cases per 100,000*	36	(Centers for Disease Control and Prevention)
*Time step for start of dose 1 rollout*^‡^, #	534, 132, 84, 74, 11	(Bluestein et al., 2021; Centers for Disease Control and Prevention, 2022; Mandavilli, 2021; Peck, 2021; Stirgus et al., 2021)
*Time step for start of dose 3 rollout*^‡^, #	N/A, 368, 323, 323, 265	(Centers for Disease Control and Prevention, 2021c; U.S. Food and Drug Administration, 2021; U.S. Food and Drug Administration, 2022a)
*Time step for start of dose 4 rollout*^‡^, #	N/A, N/A, N/A, 453, 453	(U.S. Food and Drug Administration, 2022b)
*Dose 1 vaccination rate, per day* ^‡^	0.0005, 0.0012, 0.0160, 0.0160, 0.0180	Calibrated
*Dose 2 vaccination rate, per day* ^‡^	0.010, 0.015, 0.300, 0.300, 0.450	Calibrated
*Dose 3 vaccination rate, per day* ^‡^	N/A, 0.003, 0.045, 0.038, 0.015	Calibrated
*Dose 4 vaccination rate, per day* ^‡^	N/A, N/A, N/A, 0.005, 0.008	Calibrated
*Relative risk of infection, by dose*	0.324, 0.112, 0.120, 0.120	(Barda et al., 2021; Pilishvili et al., 2021)
*Relative risk of symptoms, by dose*	0.40, 0.09, 0.09, 0.09	(Barda et al., 2021; Chung et al., 2021)
*Relative risk of hospitalization, by dose*	0.30, 0.02, 0.07, 0.07	(Barda et al., 2021; Chung et al., 2021)
*Per-dose probability of side effects*	0.18	(Chrissian et al., 2022)
*Half-life of vaccine immunity, in days*	80	(Centers for Disease Control and Prevention, 2021b)

* Values displayed for the following age groups: < 18, 18–64, and 65 + yrs.

^ Values displayed for the following age groups: < 10, 10–19, 20–29, 30–39, 40–49, 50–59, 60–69, 70–79, and 80 + yrs.

† Values displayed for the following age groups: < 1, 1–4, 5–9, 10–14, 15–19, 20–24, 25–29, 30–34, 35–39, 40–44, 45–49, 50–54, 55–59, 60–64, 65–69, 70–74, 75–79, 80–84, and 85 + yrs.

‡ Values displayed for the following age groups: < 5, 5–17, 18–49, 50–64, and 65 + yrs.

# Day 1 corresponds to 01 January 2021

**Table 2:** Proportion of adult population vaccinated, by dose and age group, for select scenarios. For each scenario, the median, 25th percentile, and 75th percentile of each outcome across 128 runs are reported below. These percentages represent coverage among the full population in each age group, not only the eligible population. Results for 18- to 49-year-olds and 50- to 64-year-olds were combined for the first two doses but treated separately for booster doses to match CDC reports and because those under 50 years of age were not eligible for the fourth dose. Green indicates higher coverage than the reference level and orange indicates lower (by at least 0.5 %).

Scenario		Ages 18–49			Ages 50–64				Ages 65+			
*Hospitalization Nudge Prob*,	*Breakthrough Nudge Prob*.	Dose 1 %	Dose 2 %	Dose 3 %	Dose 1 %	Dose 2 %	Dose 3 %	Dose 4 %	Dose 1 %	Dose 2 %	Dose 3 %	Dose 4 %
Ref. (0.102)	Ref. (0.125)	74.2 (74.1, 74.3)	61.4 (61.2, 61.5)	18.3 (18.1, 18.4)	74.2 (74.1, 74.3)	61.4 (61.2, 61.5)	36.2 (35.9, 36.4)	7.3 (7.2, 7.4)	89.8 (89.7, 90.0)	78.9 (78.7, 79.1)	51.8 (51.5, 52.2)	17.0 (16.8, 17.3)
200% of Ref. (0.204)	Ref. (0.125)	77.0 (76.9, 77.1)	64.9 (64.8, 65.0)	22.8 (22.7, 22.9)	77.0 (76.9, 77.1)	64.9 (64.8, 65.0)	40.4 (40.1, 40.7)	8.0 (7.9, 8.1)	91.0 (90.8, 91.2)	81.1 (80.9, 81.3)	55.8 (55.4, 56.2)	18.1 (17.8, 18.3)
Ref. (0.102)	50% of Ref. (0.063)	74.2 (74.1, 74.3)	61.4 (61.2, 61.5)	18.4 (18.3, 18.5)	74.2 (74.1, 74.3)	61.4 (61.2, 61.5)	36.2 (35.9, 36.6)	7.3 (7.2, 7.5)	89.8 (89.7, 90.0)	78.9 (78.7, 79.1)	Q (51.5, 52.1)	17.1 (16.8, 17.3)
Ref. (0.102)	0% of Ref.	74.2 (74.1, 74.3)	61.3 (61.2, 61.4)	18.3 (18.2, 18.5)	74.2 (74.1, 74.3)	61.3 (61.2, 61.4)	36.3 (35.9, 36.6)	7.4 (7.2, 7.5)	89.8 (89.7, 90.0)	78.9 (78.7, 79.1)	52.0 (51.5, 52.3)	17.2 (16.9, 17.4)
200% of Ref. (0.204)	50% of Ref. (0.063)	77.0 (76.9, 77.2)	64.9 (64.8, 65.0)	22.9 (22.7, 23.1)	77.0 (76.9, 77.2)	64.9 (64.8, 65.0)	40.5 (40.2, 40.8)	8.0 (7.8, 8.1)	91.0 (90.8, 91.1)	81.1 (80.8, 81.3)	55.8 (55.5, 56.1)	18.1 (17.9, 18.3)
200% of Ref. (0.204)	0% of Ref.	77.1 (76.9, 77.2)	64.9 (64.8, 65.1)	22.9 (22.8, 23.1)	77.1 (76.9, 77.2)	64.9 (64.8, 65.1)	40.6 (40.3, 40.9)	8.0 (7.8, 8.2)	91.0 (90.8, 91.1)	81.1 (80.9, 81.3)	55.9 (55.5, 56.2)	18.2 (17.9, 18.5)

**Table 3:** Overall infection rate and infections averted by end of simulation, for select scenarios. For each scenario, the median, 25th percentile, and 75th percentile of each outcome across 128 runs are reported below. Green indicates fewer infections than the reference level and orange indicates more.

Scenario		Total Infections per 100,000 PD	Infections Averted per 100,000 PD	Percent of Infections Averted	Infections Averted per Addtl. 1,000 Doses
*Hospitalization Nudge Prob*.	*Breakthrough Nudge Prob*.	*n*	*n*	%	*n*
Ref. (0.102)	Ref. (0.125)	131.1 (129.9, 132.3)	-	-	.
200% of Ref. (0.204)	Ref. (0.125)	125.8 (124.7, 127.0)	5.3 (4.0, 6.3)	4.0 (3.1, 4.8)	391.0 (296.2, 467.2)
Ref. (0.102)	50% of Ref. (0.063)	131.2 (130.1, 132.5)	−0.2 (−1.4, 1.0)	−0.1 (−1.1, 0.7)	−232.0 (−2,885.7, 2,959.4)
Ref. (0.102)	0% of Ref.	131.1 (129.9, 132.7)	0.0 (−1.6, 1.1)	0.0 (−1.2, 0.9)	1,296.0 (−1,249.6, 3,674.6)
200% of Ref. (0.204)	50% of Ref. (0.063)	125.7 (124.8, 127.2)	5.3 (3.9, 6.3)	4.1 (2.9, 4.8)	388.6 (283.9, 459.0)
200% of Ref. (0.204)	0% of Ref.	125.8 (124.4, 127.4)	5.2 (3.7, 6.6)	4.0 (2.8, 5.0)	377.9 (265.0, 477.5)

**Table 4:** Overall death rate and deaths averted by end of simulation, for select scenarios. For each scenario, the median, 25th percentile, and 75th percentile of each outcome across 128 runs are reported below. Green indicates fewer deaths than the reference level and orange indicates more.

Scenario		Total Deaths per 100,000 PD	Deaths Averted per 100,000 PD	Percent of Deaths Averted	Deaths Averted per Addtl. 1,000 Doses
*Hospitalization Nudge Prob*.	*Breakthrough Nudge Prob*.	*n*	*n*	%	*n*
Ref. (0.102)	Ref. (0.125)	0.363 (0.349, 0.384)	-	-	-
200% of Ref. (0.204)	Ref. (0.125)	0.344 (0.328, 0.362)	0.018 (0.000, 0.035)	5.1 (0.1, 9.6)	1.4 (0.0, 2.7)
Ref. (0.102)	50% of Ref. (0.063)	0.364 (0.345, 0.376)	−0.002 (−0.013, 0.018)	−0.4 (−3.6, 5.0)	9.2 (−24.7, 58.0)
Ref. (0.102)	0% of Ref.	0.360 (0.343, 0.381)	0.003 (−0.018, 0.020)	0.7 (−5.0, 5.5)	10.3 (−35.9, 57.6)
200% of Ref. (0.204)	50% of Ref. (0.063)	0.346 (0.329, 0.363)	0.017 (0.000, 0.034)	4.6 (−0.1, 9.3)	1.3 (0.1, 2.6)
200% of Ref. (0.204)	0% of Ref.	0.342 (0.330, 0.362)	0.020 (0.000, 0.033)	5.6 (0.0, 9.1)	1.4 (0.0, 2.4)

## Data Availability

All model data and code are available online at http://github.com/EpiModel/COVID-Vax-Decisions.

## Appendix A. Supporting information

Supplementary data associated with this article can be found in the online version at doi:10.1016/j.epidem.2025.100831.
